# Mucinous cystic neoplasms of the liver: current insights into epidemiology, diagnosis, and treatment

**DOI:** 10.3389/fsurg.2026.1814613

**Published:** 2026-05-08

**Authors:** Mohsin Murshid, Abdulmalik AlShamrani, Farrukh Ansari, Mohammed Saleh AlGhamdi, Saad AlHarthi, Abdulwudod M. Hefdi, Abdulaziz Rashed AlShehri, Kadi AlSayed, Faisal AlNazawi, Asma AlBarakati

**Affiliations:** 1 Department of General Surgery, Hera General Hospital, Makkah, Saudi Arabia; 2 Department of General Surgery, King Fahd General Hospital, Jeddah, Saudi Arabia; 3 Department of General Surgery, King Fahad Armed Forces Hospital, Jeddah, Saudi Arabia; 4 Department of General Surgery, Prince Mohammed Bin Nasser Hospital, Jizan, Saudi Arabia; 5 Department of General Surgery, East Jeddah General Hospital, Jeddah, Saudi Arabia; 6 Department of Surgery, International Medical Center, Jeddah, Saudi Arabia

**Keywords:** biliary cystic neoplasms, cystic liver lesions, hepatic cystic tumors, liver resection, mucinous cystic neoplasm of the liver (MCN-L), ovarian-type stroma

## Abstract

Mucinous cystic neoplasms of the liver (MCN-L) are rare cyst-forming epithelial tumors defined by mucin-producing epithelium and characteristic ovarian-type stroma. Once grouped with other biliary cystic lesions, MCN-L are now recognized as a distinct premalignant entity with potential for progression to invasive carcinoma. Accurate preoperative diagnosis remains difficult because imaging findings frequently overlap with those of other cystic liver lesions, including simple hepatic cysts, hydatid cysts, and intraductal papillary neoplasms of the bile duct. Laboratory markers, cyst fluid analysis, and biopsy have limited diagnostic reliability and cannot confidently exclude malignancy. Given the risk of malignant transformation and diagnostic uncertainty, complete surgical excision is recommended for all suspected MCN-L irrespective of symptom status or lesion size. Resection with negative margins is associated with excellent long-term outcomes, whereas non-definitive procedures such as cyst fenestration or drainage are associated with high recurrence rates. In selected cases, intraoperative findings—particularly the presence of the “peeling sign”—may allow safe parenchyma-sparing excision while preserving functional liver tissue. Prognosis is generally favorable in non-invasive disease but significantly worse when invasive carcinoma is present, a feature that cannot be reliably predicted before surgery. This review synthesizes current evidence on the epidemiology, diagnostic approach, imaging characteristics, pathology, surgical management, and prognosis of MCN-L, and proposes a practical framework for the evaluation and management of cystic liver lesions suspicious for MCN-L.

## 1 Introduction

Mucinous cystic neoplasms of the liver (MCN-L) are rare cystic hepatic tumors. These lesions account for less than 5% of all hepatic cysts in population-based studies but represent approximately 10% of hepatic cysts encountered in surgical series, reflecting referral and selection bias toward complex or symptomatic lesions (1, 2). MCN-L are defined by a mucin-producing epithelial lining supported by characteristic ovarian-type stroma. They are clinically important because of their premalignant potential, as some lesions may harbor high-grade dysplasia or invasive carcinoma. Consequently, suspected MCN-L are generally managed with complete surgical excision (1, 3, 4).

Historically, the terms *biliary cystadenoma* and *biliary cystadenocarcinoma* were widely used but applied inconsistently. Many earlier reports grouped biologically distinct entities together, most notably failing to distinguish MCN-L from intraductal papillary neoplasms of the bile duct (IPNB). IPNB are ductal tumors that typically communicate with the biliary tree and demonstrate distinct clinical behavior and management strategies (1, 5). Re-evaluation of historical cohorts has revealed substantial misclassification. In one surgical series, approximately one quarter of previously classified cases lacked ovarian-type stroma on pathologic re-examination, while nearly 80% of true MCN cases had not been originally recognized as such (6).

The modern World Health Organization (WHO) classification has clarified this diagnostic ambiguity by defining MCN-L as cyst-forming epithelial tumors that typically lack communication with the bile ducts and contain mucinous epithelium supported by ovarian-type subepithelial stroma (7, 8). Adoption of this definition has improved consistency in both pathologic reporting and clinical decision-making.

Despite advances in imaging and pathology, preoperative diagnosis remains challenging. Radiologic features such as multiloculated cystic lesions with internal septations or mural nodules may raise suspicion for MCN-L; however, substantial overlap exists with other cystic liver diseases, and invasive disease cannot be reliably excluded preoperatively (5, 9). This diagnostic uncertainty underlies the central role of surgery in management. Incomplete procedures such as aspiration, drainage, or fenestration still occur in clinical practice and are associated with high recurrence rates, reinforcing recommendations for definitive resection when MCN-L is suspected (3, 10).

Recent literature has emphasized improved preoperative classification, clearer radiologic distinction between MCN-L and IPNB, and increasing use of minimally invasive liver surgery in selected patients, supported by advances in perioperative care and imaging-based surgical planning (5, 9). This review provides a clinically oriented synthesis of current evidence on mucinous cystic neoplasms of the liver, integrating imaging characteristics, pathologic criteria, and surgical decision-making. Particular attention is given to practical management strategies, including emerging intraoperative concepts, which may assist in guiding parenchyma-sparing surgical approaches for selected lesions.

## 2 Methods

A structured literature search was conducted in PubMed/MEDLINE from database inception through December 2025, supplemented by searches in Google Scholar, ResearchGate, and manual review of reference lists.

Study selection was performed by the authors through a structured screening process. Duplicates were removed using reference manager Zotero. Studies were selected based on predefined criteria including clinical relevance, methodological quality, and contribution to key domains (epidemiology, diagnosis, imaging, pathology, and management). Studies focusing exclusively on non-hepatic disease, unrelated cystic lesions, experimental models, or non–data-driven publications were excluded. Data were extracted on study characteristics, diagnostic features, imaging findings, histopathology, surgical management, and outcomes, and synthesized qualitatively. The detailed search is summarized in Table 1.

**Table 1:** Summary of literature search methodology, including databases searched, predefined search strategy, timeframe (inception to December 2025), screening.

Source	Search strategy	Timeframe	Screening approach	Records identified	Included studies
PubMed/MEDLINE	(“mucinous cystic neoplasm liver” OR “biliary mucinous cystic neoplasm” OR “hepatic mucinous cystic neoplasm” OR “biliary cystadenoma” OR “biliary cystadenocarcinoma” OR (“mucinous cystic neoplasm”[Title/Abstract] AND liver[Title/Abstract])) AND (“ovarian-type stroma” OR “ovarian-like stroma”) AND (“imaging” OR “radiology” OR “MRI” OR “CT” OR “ultrasound” OR “diagnosis” OR “pathology” OR “histopathology” OR “surgical management” OR “liver resection”)	Inception – December 2025	Title/abstract screening → full-text review	80	52
Google Scholar	mucinous cystic neoplasm liver biliary cystadenoma ovarian stroma imaging surgery	Up to December 2025	Manual screening - first 20 pages	—	—
ResearchGate	mucinous cystic neoplasm liver	Up to December 2025	Manual screening - first 10 pages	—	—
Secondary search	Reference lists of included studies	—	Manual screening	—	—

## 3 Epidemiology

Mucinous cystic neoplasms of the liver (MCN-L) are rare lesions that represent a small proportion of cystic hepatic tumors. Epidemiologic characterization has historically been challenging because these lesions were previously grouped under the broader term “biliary cystadenoma,” a category that included heterogeneous entities such as intraductal neoplasms, hamartomas, congenital cysts, and polycystic liver disease (11, 12). As a result, reliable estimates of incidence and risk factors were difficult to establish. Prior to 2005, fewer than 200 cases of hepatobiliary cystadenomas had been documented in the literature, increasing to approximately 250 reported cases a decade later, highlighting both the rarity of these tumors and the limited availability of population-level data (13, 14).

A defining epidemiologic feature of MCN-L is their marked female predominance. When ovarian-type stroma—the key histopathologic criterion introduced in modern classification—is used as a mandatory diagnostic requirement, these tumors occur almost exclusively in women (96%–100%) (11, 15, 16). Male patients are exceptionally rare, and reported cases should be interpreted cautiously, as many likely represent misclassified lesions or alternative cystic hepatobiliary tumors (3, 17). This sex distribution closely parallels pancreatic mucinous cystic neoplasms, which also demonstrate a strong female predominance (18).

### 3.1 Impact of ovarian-type stroma and WHO reclassification

The recognition of ovarian-type stroma as a defining diagnostic feature significantly reshaped the epidemiologic understanding of these tumors. Early studies did not systematically assess this feature. For example, a Mayo Clinic series published in 1974 reported cystadenomas in 2.2% of grossly detectable hepatic cysts; however, the absence of ovarian-like stromal evaluation limits the applicability of these findings under current classification systems (19, 20). In a landmark Cleveland Clinic study, ovarian-type stroma was identified in 56% of benign cystadenomas and 50% of malignant cases, with all stromal-positive tumors occurring in women and malignant cases presenting at a higher mean age (13).

In 2010, the World Health Organization formally reclassified hepatobiliary cystic tumors containing ovarian-type stroma as mucinous cystic neoplasms, stratified by dysplasia grade or associated invasive carcinoma, thereby establishing a biologically consistent diagnostic framework (21). This reclassification separated MCN from intraductal papillary neoplasms of the bile duct (IPNB), allowing more accurate epidemiologic assessment.

### 3.2 Contemporary incidence and geographic patterns

Following WHO reclassification, MCNs have been reported in approximately 10.5%–11% of resected hepatobiliary cysts in surgical series, although large population-based cohorts report lower rates of approximately 0.6–2.2%, likely reflecting referral patterns and differences in case selection (2, 22–24). Malignant transformation is uncommon but has been associated with older patient age at diagnosis, with malignant cases typically presenting in the sixth decade of life compared with earlier presentation of benign disease (57–64 vs. 41–44 years) (11, 15, 16).

Geographic differences in the distribution of hepatobiliary cystic neoplasms have also been reported. In South Korean cohorts, intraductal papillary neoplasms of the bile duct occur approximately 3.5 times more frequently than MCN, suggesting regional variation in disease patterns or diagnostic practices compared with Western populations (23).

The available evidence indicates that MCN-L are rare but distinct cystic hepatic tumors characterized by a strong female predominance and typically presenting in middle age. The introduction of ovarian-type stroma as a defining diagnostic criterion and the subsequent WHO reclassification have significantly improved epidemiologic clarity by separating MCN-L from other cystic biliary tumors, particularly IPNB. For clinicians, the key implication is that a multilocular cystic liver lesion in a middle-aged woman should raise strong suspicion for MCN-L, particularly when imaging demonstrates internal septations or mural nodules. Because reliable epidemiologic risk stratification remains limited and malignant transformation cannot be predicted preoperatively, management decisions should rely primarily on radiologic features and clinical suspicion rather than demographic factors alone.

## 4 Pathogenesis

Mucinous cystic neoplasms of the liver (MCN-L) arise almost exclusively within the liver and typically present as solitary intrahepatic lesions. Extrahepatic mucinous cystic neoplasms of the biliary system are exceedingly rare. There is no consistent predilection for a specific hepatic segment, although most lesions are large at the time of diagnosis (14, 25). Several series have reported a marked predominance in the left hepatic lobe, with more than 70% of tumors arising in this location despite its smaller volume compared with the right lobe (23, 26). The basis for this preferential localization remains uncertain. One proposed explanation is that ovarian-type stroma may recapitulate periductal fetal mesenchyme, which could be relatively more prominent in the left liver because of its embryologic proximity to the pancreatobiliary tract (23, 26, 27).

### 4.1 Hormonal and stromal mechanisms

No consistent modifiable risk factors have been identified for MCN-L. Tobacco use, alcohol consumption, cirrhosis, viral hepatitis, and hepatobiliary stone disease have each been reported infrequently, generally in less than 15% of cases and often below 5% (22, 23). The pathogenesis of MCN-L is closely linked to the presence of ovarian-type stroma, which represents a defining diagnostic feature rather than an incidental histologic finding. Immunohistochemical studies consistently demonstrate estrogen receptor and progesterone receptor expression within stromal cells, supporting a hormonally influenced mechanism (16, 28).

Several hypotheses have been proposed to explain tumor origin. One theory suggests development from ectopic embryonic tissue with Müllerian differentiation, whereas another proposes origin from peribiliary glands capable of mucinous transformation under hormonal influence. Although no single hypothesis fully explains all cases, the hormonal theory is supported by epidemiologic, histologic, and molecular observations (28, 29).

### 4.2 Malignant potential and progression

MCN-L possess recognized malignant potential, with reported rates of invasive carcinoma ranging from 5% to 15%. Precise estimates remain uncertain because most available data derive from surgically treated cohorts. Several clinicopathologic features have been associated with increased malignant risk, including larger tumor size, mural nodules, papillary projections, and high-grade epithelial atypia (13, 14, 17). Current evidence supports a stepwise progression model from low-grade dysplasia to high-grade dysplasia and ultimately invasive carcinoma, mirroring the neoplastic progression observed in pancreatic mucinous cystic neoplasms.

Importantly, the degree of dysplasia cannot be reliably predicted before surgery. Neither imaging characteristics nor clinical features consistently distinguish low-grade from high-grade disease. This diagnostic uncertainty reinforces the recommendation for complete surgical excision once MCN-L is suspected (13, 17, 30).

**Figure 1:**
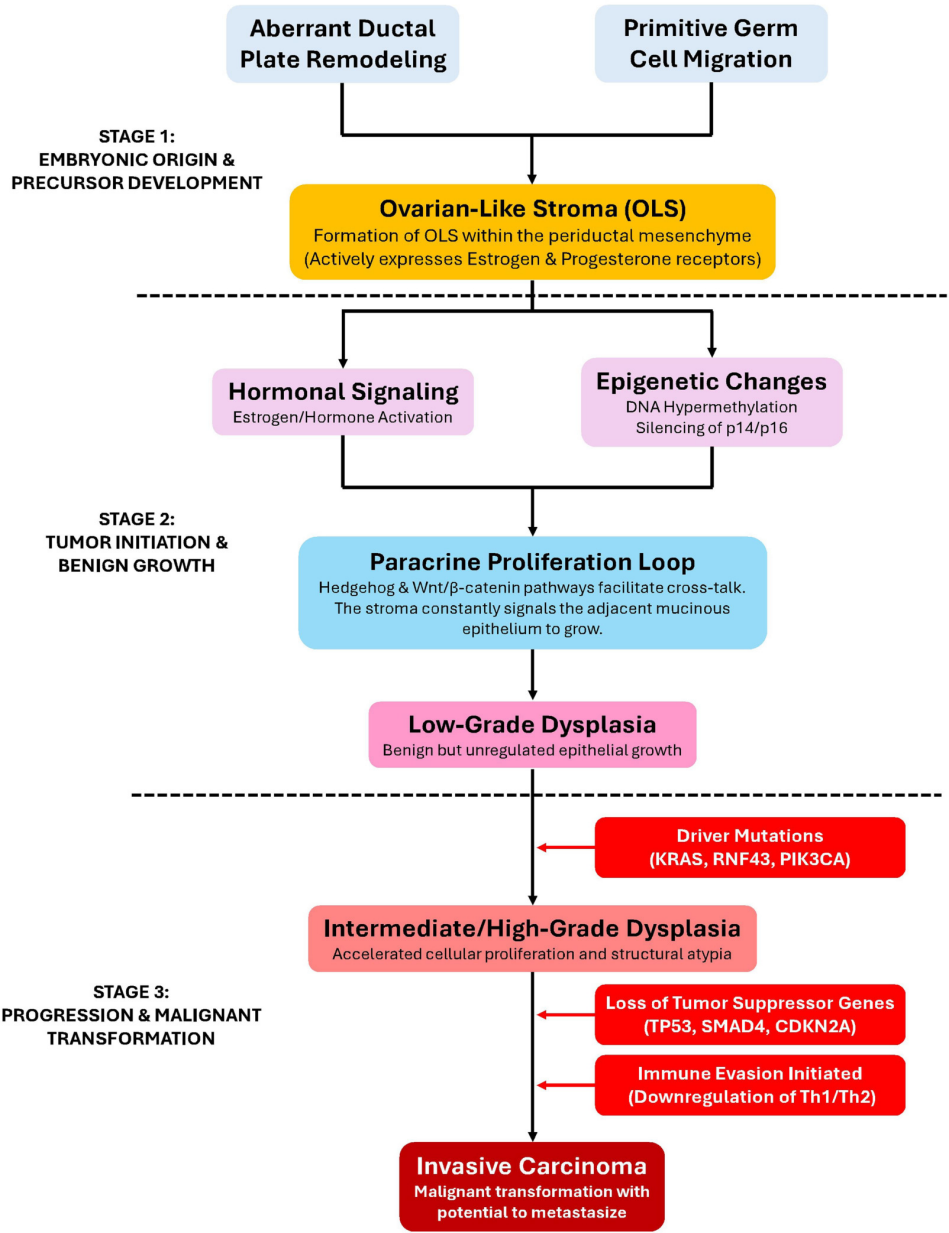
Proposed molecular pathogenesis and step-wise progression pathway of mucinous cystic neoplasm of the liver (MCN-L) (31–36). CDKN2A (p16), cyclin-dependent kinase inhibitor 2A; KRAS, Kirsten rat sarcoma viral oncogene homolog; MCN-L, mucinous cystic neoplasm of the liver; OLS, ovarian-like stroma; PIK3CA, phosphatidylinositol-4,5-bisphosphate 3-kinase catalytic subunit alpha; RNF43, ring finger protein 43; SMAD4, SMAD family member 4; Th1/Th2, type 1/type 2 T-helper cells; TP53, tumor protein p53; Wnt, wingless-related integration site.

In Summary, current evidence suggests that MCN-L arise through hormonally influenced stromal–epithelial interactions, with ovarian-type stroma playing a central role in tumor development and classification. The marked female predominance and expression of estrogen and progesterone receptors support a hormonally mediated pathogenesis, although the precise cellular origin remains uncertain. From a clinical perspective, the key implication is that malignant potential exists even in lesions that appear radiologically benign, and the degree of dysplasia cannot be reliably predicted before resection. Consequently, the biological behavior of MCN-L reinforces the current management paradigm in which surgical excision is recommended once the diagnosis is suspected, rather than relying on prolonged surveillance or invasive diagnostic attempts to exclude malignancy. The proposed stepwise molecular pathogenesis of MCN-L is illustrated in Figure 1 (31–36).

## 5 Pathology and classification

The World Health Organization (WHO) classification has standardized the terminology and conceptual framework for mucinous cystic neoplasms of the liver (MCN-L). In the current WHO framework, MCN-L are categorized into three groups: (i) MCN with low-grade dysplasia, (ii) MCN with high-grade dysplasia, and (iii) MCN with associated invasive carcinoma. This classification replaces earlier terminology such as *biliary cystadenoma* and *biliary cystadenocarcinoma*. Lesions previously described as cystadenocarcinoma are now recognized as invasive carcinomas arising within MCN-L, reflecting a continuous disease spectrum rather than separate benign and malignant entities (34, 37).

This reclassification has important conceptual and clinical implications. It establishes MCN-L as a premalignant condition with the potential for progression across a spectrum of dysplastic changes rather than representing distinct benign and malignant tumors. Even lesions with low-grade dysplasia carry a risk of progression, supporting recommendations for definitive surgical resection once MCN-L is suspected (28, 34).

Histopathology remains essential for definitive diagnosis because clinical features and imaging alone cannot reliably distinguish MCN-L from other cystic hepatic lesions. The diagnosis is established after examination of the resected specimen and requires three key criteria: (i) a cystic lesion lined by mucin-producing epithelium, (ii) absence of communication with the bile ducts, and (iii) the presence of ovarian-type stroma beneath the epithelial lining (16, 28, 34, 37). Gross examination typically reveals large multilocular cystic lesions with a mean diameter of approximately 11 cm, surrounded by a fibrous capsule and containing mucinous or hemorrhagic fluid (24, 38).

Microscopically, the epithelial lining consists of columnar or cuboidal mucin-producing cells. The degree of epithelial atypia may vary within a single lesion, with areas of low-grade dysplasia coexisting alongside high-grade dysplasia or invasive carcinoma. This intralesional heterogeneity limits the diagnostic value of preoperative biopsy or small tissue samples, as these may fail to capture the highest-grade component of the lesion (11, 28).

### 5.1 Ovarian-type stroma

Ovarian-type stroma is the defining histologic feature of MCN-L and may be focal rather than diffuse. Similarly, mucinous epithelium may be absent or focal in some areas, emphasizing the need for extensive sampling to establish the diagnosis and exclude invasive carcinoma (26, 39). The stroma consists of densely packed spindle-shaped cells resembling ovarian tissue and typically demonstrates immunohistochemical positivity for estrogen receptor and progesterone receptor. Inhibin and calretinin expression may also be observed in some cases (16, 33, 37). Key diagnostic histopathological and immunohistochemical findings supporting the diagnosis of MCN-L are illustrated in Figure 3.

### 5.2 Molecular features

Molecular data on MCN-L remain limited. In the first comparative genetic study of hepatic and pancreatic mucinous cystic neoplasms, KRAS, GNAS, RNF43, and PIK3CA alterations were evaluated. KRAS mutations were identified in approximately 20% of cases and were associated with intermediate- and high-grade dysplasia rather than low-grade lesions, suggesting a role in tumor progression rather than initiation. Identical KRAS mutations were observed in both low- and higher-grade areas within the same tumor, supporting a model of clonal progression. KRAS-mutated tumors more frequently demonstrated multilocular architecture and increased expression of mucin proteins including MUC1, MUC2, and MUC5AC. In contrast, GNAS, RNF43, and PIK3CA mutations were not detected in analyzed hepatic MCN-L cases. KRAS alterations also appear less frequent in hepatic MCN-L than in pancreatic mucinous cystic neoplasms, which may partly explain the lower malignant potential observed in MCN-L. Alterations in hedgehog signaling pathways, including SMO mutations and GLI1 overexpression, have also been reported and support a relatively indolent molecular profile in hepatic MCN (33, 38). The key histopathologic and ancillary features differentiating MCN-L from other cystic hepatic lesions are summarized in Table 2.

**Table 2:** Histopathologic, immunohistochemical, and selected imaging features differentiating mucinous cystic neoplasms of the liver from other cystic hepatic lesions.

Neoplasm/Lesion	MCN	IPNB	Simple biliary cyst	Endometrial cyst	Ciliated foregut cyst	Hydatid cyst	Caroli disease	BilIN
Unifocal cyst on imaging	+	+/-	+/-	+/-	+	+	-	-
Septations on imaging	+	+/-	-	-	-	+/-	+/-	-
Contiguous with biliary tree	-	+	+/-	-	-	-	+	+
CD10	-	-	-	+ (s)	-	-	-	-
Spindled stroma	+	-	-	+	-	-	-	-
ER, PR	+ (s)	-	-	+ (s,e)	-	-	-	-
Inhibin	+ (s)	-	-	-	-	-	-	-
Mucin stains	+	+/-	-	-	+(g)	-	-	+/-

Reproduced with permission from Valasek et al. (40), Wolters Kluwer Health, Inc., via Copyright Clearance Center. BilIN, biliary intraepithelial neoplasia; ER, estrogen receptor; PR, progesterone receptor; s, stromal expression; e, epithelial expression; g, goblet cells.

The current pathologic framework establishes MCN-L as a distinct premalignant cystic tumor defined by the presence of ovarian-type stroma and mucin-producing epithelium. Because these defining features can only be confirmed histologically, definitive diagnosis is typically established after surgical resection rather than before intervention. Furthermore, intralesional heterogeneity and the limited reliability of biopsy or cyst fluid sampling make preoperative grading of dysplasia difficult. From a clinical standpoint, these limitations reinforce the principle that management decisions should rely primarily on radiologic suspicion rather than histologic confirmation, and suspected MCN-L should be treated with complete surgical excision to allow definitive diagnosis and prevent progression to invasive carcinoma.

## 6 Clinical presentation

Most patients with mucinous cystic neoplasms of the liver (MCN-L) are asymptomatic at presentation, and lesions are frequently detected incidentally during imaging performed for unrelated indications. This is particularly true for small- to moderate-sized tumors (1, 3). MCN-L predominantly affect middle-aged women, although rare cases have been reported in adolescents. In one such report, a cyst increased in size from 35 mm to 75 mm over eight months, illustrating that rapid tumor growth may occur even in younger patients and should prompt consideration of early surgical intervention (41).

### 6.1 Symptoms

When symptoms occur, they are generally nonspecific and related primarily to tumor size rather than biologic aggressiveness. Abdominal pain or discomfort is the most common presenting complaint, followed by abdominal fullness, early satiety, or a palpable mass (3, 25). Jaundice is uncommon in MCN-L and should prompt careful reassessment of the diagnosis. When present, it may result from extrinsic bile duct compression by a large lesion or suggest an alternative diagnosis such as intraductal papillary neoplasm of the bile duct (IPNB), as true biliary communication is not a characteristic feature of MCN-L (5, 29).

### 6.2 Natural history and acute complications

The natural history of untreated MCN-L remains poorly defined because prospective observation studies are rarely feasible. Indirect evidence from historical reports and cases of recurrence after incomplete treatment suggests progressive tumor growth over time. Lesions managed with aspiration, drainage, or fenestration almost invariably recur (13, 42). Acute complications are uncommon but have been reported, including intracystic hemorrhage, infection, and spontaneous rupture. These events may present with acute abdominal pain or fever and can mimic liver abscess or hemorrhagic cysts on imaging (25, 43). Despite these occasional complications, most MCN-L follow a relatively slow-growing course.

### 6.3 Risk of malignancy

Malignant transformation remains an important concern in MCN-L. Invasive carcinoma arising within these lesions has been documented in multiple series, although reported frequencies vary (3, 13, 14). Clinical presentation does not reliably correlate with histologic grade; patients with low-grade dysplasia and those with invasive carcinoma may present with similar symptoms or remain asymptomatic. Tumor size is also an imperfect predictor of malignancy, as large lesions may harbor low-grade disease while smaller lesions may demonstrate high-grade dysplasia (1, 14).

### 6.4 Clinical approach

Given the uncertain natural history and the inability to reliably exclude malignancy before surgery, observation alone is generally not recommended when MCN-L is suspected. Current evidence supports early referral for surgical evaluation. Complete surgical excision provides excellent long-term outcomes and minimizes the risks of recurrence and malignant progression (3, 13).

The available evidence indicates that clinical presentation alone is not a reliable indicator of disease severity in MCN-L. Many patients remain asymptomatic, while symptomatic presentation is usually related to tumor size rather than malignant potential. Importantly, neither symptom burden nor tumor size can reliably predict the presence of high-grade dysplasia or invasive carcinoma. For clinicians, the key implication is that incidental multilocular cystic liver lesions suspicious for MCN-L should not be managed conservatively solely on the basis of minimal symptoms or stable clinical status. Instead, early referral for surgical evaluation is recommended once radiologic features suggest MCN-L, as definitive diagnosis and treatment can only be achieved through complete surgical excision.

## 7 Differential diagnosis of cystic liver lesions

The differential diagnosis of cystic liver lesions is broad, and many entities share overlapping imaging features with mucinous cystic neoplasms of the liver (MCN-L). Accurate differentiation is important because management strategies differ substantially. In many cases, a definitive diagnosis is established only after surgical excision and histologic examination (1, 3).

### 7.1 Simple hepatic cysts

Simple hepatic cysts are the most common cystic lesions of the liver. They are benign, often asymptomatic, and typically appear as unilocular, thin-walled cysts without septations or mural nodules, with no post-contrast enhancement (43). In contrast, MCN-L are usually multiloculated and contain internal septations. However, early or small MCN-L may resemble simple cysts, leading to misdiagnosis and inappropriate treatment such as fenestration (3, 13).

Detailed analysis of septation morphology has shown that septations arising directly from the cyst wall without external indentation are highly sensitive for MCN-L, whereas septations originating from external macrolobulations are highly specific for simple hepatic cysts (44). Key differentiating imaging features are summarized in Table 3.

**Table 3:** Imaging features that may help differentiate mucinous cystic neoplasms of the liver (MCN-L) from simple hepatic cysts, with particular emphasis on septation origin and enhancement patterns (44).

Imaging feature	Diagnostic interpretation	Strength of association
Septations arising from the cyst wall without external indentation	Strongly favors MCN-L	Highly sensitive
Septations arising from external macrolobulations	Favors simple hepatic cyst	Highly specific
Septal enhancement on MRI	Supports MCN-L but not diagnostic	High sensitivity, limited specificity
Predominant left-lobe localization	Supports MCN-L	Supportive
Biliary duct dilatation	Minor worrisome feature	Low specificity

### 7.2 Hydatid disease

Hydatid cysts, caused by *Echinococcus* infection, remain common in endemic regions. Imaging may demonstrate daughter cysts, calcifications, or detached membranes, and serologic testing may support the diagnosis (45). Despite these characteristic features, hydatid disease can occasionally mimic MCN-L. Conversely, MCN-L has sometimes been misdiagnosed as hydatid cysts, resulting in delayed definitive surgical management. Careful clinical history and serologic evaluation are therefore essential (25, 45).

### 7.3 Liver abscess

Liver abscesses typically present with fever, abdominal pain, and laboratory evidence of infection. Imaging often shows thick-walled cystic lesions with surrounding inflammatory changes, and gas may be present in some cases (43). MCN-L rarely present with infection; however, intracystic infection can occur and may mimic an abscess on imaging. Failure to respond to antibiotic therapy should prompt reconsideration of the diagnosis (14, 25).

### 7.4 Polycystic liver disease

Polycystic liver disease is characterized by multiple hepatic cysts and is frequently associated with autosomal dominant polycystic kidney disease. The cysts are generally uniform and lack septations or mural nodules (10). In contrast, MCN-L are almost always solitary lesions, and diffuse cystic involvement strongly favors polycystic liver disease rather than MCN-L (3, 10).

### 7.5 Cystic metastases

Certain malignancies, including neuroendocrine tumors and ovarian cancers, may produce cystic liver metastases. These lesions often occur in patients with a known primary malignancy and may demonstrate irregular walls or peripheral enhancement (10, 43). Clinical history is therefore critical in interpretation. Although imaging findings may be suggestive, distinction from MCN-L is not always possible before surgery (46).

### 7.6 Intraductal papillary neoplasm of the bile duct

Intraductal papillary neoplasm of the bile duct (IPNB) represents the most important differential diagnosis. IPNB are mucin-producing ductal tumors that communicate with the biliary tree and frequently result in bile duct dilatation and mucin secretion (37, 47). On imaging, IPNB may appear cystic and multiloculated, closely resembling MCN-L. Magnetic resonance cholangiopancreatography is particularly useful in demonstrating ductal communication. Histologically, IPNB lack ovarian-type stroma. This distinction is critical because IPNB demonstrate different growth patterns and require different surgical strategies (37, 47, 48). The key clinicopathologic and radiologic differences between mucinous cystic neoplasms of the liver and intraductal papillary neoplasms of the bile duct are summarized in Table 4 (1).

**Table 4:** Comparison of mucinous cystic neoplasms of the liver (MCN-L) and intraductal papillary neoplasms of the bile duct (IPNB), highlighting key radiologic, histopathologic, and immunophenotypic features relevant for differential diagnosis.

Feature	Mucinous cystic neoplasm of the liver (MCN-L)	Intraductal papillary neoplasm of the bile duct (IPNB)
Radiographic findings	Multilocular cystic lesion, often with internal septations or “cyst-in-cyst” appearance	Multicystic, grape-like lesions with papillary nodules and associated bile duct dilatation
Bile duct communication	Usually absent	Present
Stroma	Ovarian-type stroma; ER+, PR+	Fibrous stroma; ER−, PR−
Epithelial markers	MUC5AC−, CK7+, CK20−, MUC2−, MUC6−	MUC5AC+, variable CK7, CK20, MUC2, MUC6
Malignant potential	Generally low	Higher, with frequent progression to invasive carcinoma

MCN-L, mucinous cystic neoplasm of the liver; IPNB, intraductal papillary neoplasm of the bile duct; ER, estrogen receptor; PR, progesterone receptor; CK7, cytokeratin 7; CK20, cytokeratin 20; MUC, mucin; (+) positive expression; (−) negative expression.

Adapted from Hutchens et al. (1), licensed under the Creative Commons Attribution–NonCommercial 3.0 (CC BY-NC 3.0) license, Dove Medical Press.

Differentiating MCN-L from other cystic liver lesions relies on integrating imaging findings with clinical context rather than depending on a single diagnostic feature. Features such as multiloculation, internal septations, mural nodules, and the absence of biliary communication increase suspicion for MCN-L, whereas diffuse cystic disease, clear infectious features, or typical hydatid morphology favor alternative diagnoses. However, considerable overlap remains, and no imaging characteristic can definitively exclude MCN-L. The key practical implication is that when imaging findings are indeterminate, but MCN-L cannot be confidently ruled out, surgical consultation should be considered rather than prolonged diagnostic observation, given the premalignant nature of the lesion and the high recurrence rates associated with incomplete treatment.

The key clinical, radiologic, and management features distinguishing MCN-L from other cystic liver lesions are summarized in Table 5.

**Table 5:** Key clinical, radiologic, and management features help differentiate mucinous cystic neoplasms of the liver from other cystic liver lesions encountered in clinical practice.

Condition	Typical patient profile	Imaging appearance	Bile duct communication	Key distinguishing clues	Usual management
MCN-L	Predominantly women, 40–60 years	Solitary cyst, often multiloculated, internal septations ± mural nodule	Absent (typical)	Premalignant lesion; ovarian-type stroma on pathology	Complete surgical excision
Simple hepatic cyst	Any sex; often incidental	Unilocular thin-walled cyst without septations or nodules	Absent	No enhancement, no internal complexity	Observation; fenestration if symptomatic
Hydatid cyst	Endemic regions; exposure history	Multiloculated cyst with daughter cysts, membranes, calcification	Absent	Positive serology; characteristic daughter cysts	Antiparasitic therapy ± surgery
Liver abscess	Febrile patient; elevated WBC/CRP	Thick-walled lesion with rim enhancement and surrounding edema	Variable	Clinical sepsis; response to antibiotics/drainage	Antibiotics ± percutaneous drainage
Polycystic liver disease	Often familial; renal cysts	Multiple cysts diffusely involving the liver	Absent	Diffuse distribution rather than solitary lesion	Symptom-based management
Cystic metastases	Known primary malignancy	Multiple cystic lesions, often with rim enhancement	Absent	History of malignancy; extrahepatic disease	Systemic therapy ± selected surgery

MCN-L, mucinous cystic neoplasm of the liver; IPNB, intraductal papillary neoplasm of the bile duct; WBC, white blood cell count; CRP, C-reactive protein.

## 8 Imaging

Imaging is central to the evaluation of suspected mucinous cystic neoplasms of the liver (MCN-L). Most lesions are initially detected on ultrasound or cross-sectional imaging performed for unrelated indications. Imaging defines lesion size, internal architecture, and relationships to surrounding structures but cannot provide a definitive diagnosis (3, 5). Even in experienced centers, preoperative radiologic diagnosis remains unreliable. In one surgical series, accurate preoperative identification of a hepatobiliary cystic neoplasm was achieved in only about half of cases, and in nearly one third a neoplastic process was not suspected (1, 3). Because of these limitations, imaging findings should be interpreted in clinical context and used primarily to guide surgical planning rather than determine eligibility for surgery. Current evidence supports surgical excision based on suspicion alone rather than radiologic certainty (1, 3).

### 8.1 General cross-sectional imaging features

On cross-sectional imaging, MCN-L most commonly demonstrate loculations (84.2%) and septations (63.2%), while malignant lesions more frequently show multiloculation (56.9%), mural nodularity (16.5%), and biliary ductal dilatation (17.7%) (22). Although MCN-L are typically multilocular, approximately 6%–10% may appear unilocular (14, 24, 44, 49, 50). MCN-L usually demonstrate multiloculated cysts with septations or a cyst-in-cyst appearance and rarely contain mural nodules unless malignant. In contrast, intraductal papillary neoplasms of the bile duct (IPNB) often present as multicystic, grape-like lesions with papillary nodules and peripheral bile duct dilatation (23). Several systematic reviews have also demonstrated a strong predilection for MCN-L to arise in the left hepatic lobe, particularly segment IV, with 69%–76% of lesions occurring in this location (6, 23, 28, 51).

### 8.2 Imaging features suggesting aggressive behavior

Most benign MCN-L are thin-walled, whereas rim thickening or contrast enhancement raises concern for malignant transformation, particularly when associated with irregular wall thickness or papillary projections (52, 53). Degenerative changes such as hemorrhage or fibro-inflammatory nodules may produce mass-like appearances and raise radiologic suspicion for malignancy even in non-carcinomatous lesions (6). Traditional radiologic features associated with malignant MCN-L demonstrate limited predictive value, with reported sensitivity of 81% but specificity of only 21%, highlighting the difficulty of preoperative risk stratification based on imaging alone (6). Imaging features associated with more aggressive behavior and increased surgical complexity are summarized in Table 6.

**Table 6:** Imaging findings that should raise concern for aggressive behavior in mucinous cystic neoplasms of the liver.

Finding	Importance	Practical interpretation
Mural nodule (enhancing)	Linked with higher-grade disease in many cystic tumors	Treat as higher-risk → plan oncologic resection
Thick/irregular wall	Suggests aggressive biology or inflammation	Increases suspicion; avoid limited procedures
Solid component	Raises concern for invasion	Manage as potentially malignant
Rapid growth (documented)	Suggests neoplastic behavior	Strengthens indication for resection
Restricted diffusion (MRI)	Can correlate with cellular tissue	Supportive, not definitive
Biliary dilatation/duct communication	Suggests IPNB rather than MCN-L	Reframe diagnosis; plan duct-focused surgery

These features are not diagnostic of malignancy but may influence surgical planning and the extent of resection. MCN-L, mucinous cystic neoplasm of the liver; MRI, magnetic resonance imaging.

### 8.3 Structured imaging criteria and risk stratification

A retrospective blinded study demonstrated that septations arising directly from the internal cyst wall without external indentation significantly improved differentiation of biliary cystadenomas from benign hepatic cysts (54). This morphologic criterion was subsequently validated using the 2010 WHO definition of MCN, demonstrating high sensitivity but modest specificity (56.3%) (44).

The 2022 European Association for the Study of the Liver (EASL) Clinical Practice Guidelines introduced an imaging-based risk stratification system for MCN-L, classifying lesions as high or low suspicion based on combinations of major and minor worrisome features identified on cross-sectional imaging [Table 7 (55)]. Despite these structured approaches, diagnostic performance remains limited, with modest sensitivity and variable specificity across imaging-based strategies (44, 55, 56). The diagnostic performance of commonly used imaging modalities is summarized in Table 8.

**Table 7:** Imaging features used to stratify the likelihood of mucinous cystic neoplasms of the liver according to the European Association for the Study of the Liver (EASL) recommendations.

Category	Imaging feature
Major features	Mural nodularity
	Thick septations (>3 mm)
	Internal hemorrhage
Minor features	Upstream biliary dilatation
	Thin septations (<3 mm)
	Perilesional perfusion changes
	Fewer than three co-existing hepatic cysts

High suspicion is defined by the presence of at least one major and one minor feature (10, 49). MCN-L, mucinous cystic neoplasms of the liver.

**Table 8:** Diagnostic performance of imaging modalities for mucinous cystic neoplasms of the liver (MCN-L).

Diagnostic method	Sensitivity	Specificity
EASL imaging criteria	∼40%	∼80%
CT (routine interpretation)	46.1%	—
MRI (routine interpretation)	57.1%	—
MRI septal enhancement	100%	52.9%

Sensitivity and specificity are reported where available. MCN-L, mucinous cystic neoplasm of the liver; EASL, European Association for the Study of the Liver; CT, computed tomography; MRI, magnetic resonance imaging.

### 8.4 Ultrasound

Ultrasound is frequently the initial imaging modality and can achieve up to 90% sensitivity in characterizing suspected MCN-L, which typically present as single, large cystic lesions with a mean diameter of approximately 11 cm (range 5–23 cm) (57). MCN-L usually appear as well-defined cystic lesions with multiloculation and internal septations. Cyst contents may appear anechoic or demonstrate low-level internal echoes due to mucin or debris. Thick, uneven cyst walls and echogenic septa are common, while mural nodules—when present—raise concern for malignancy (12, 58). Despite its utility, ultrasound is operator-dependent and may not reliably detect mural nodules or subtle wall irregularities, necessitating further evaluation with CT or MRI (1, 43).

### 8.5 Computed tomography

**Figure 2:**
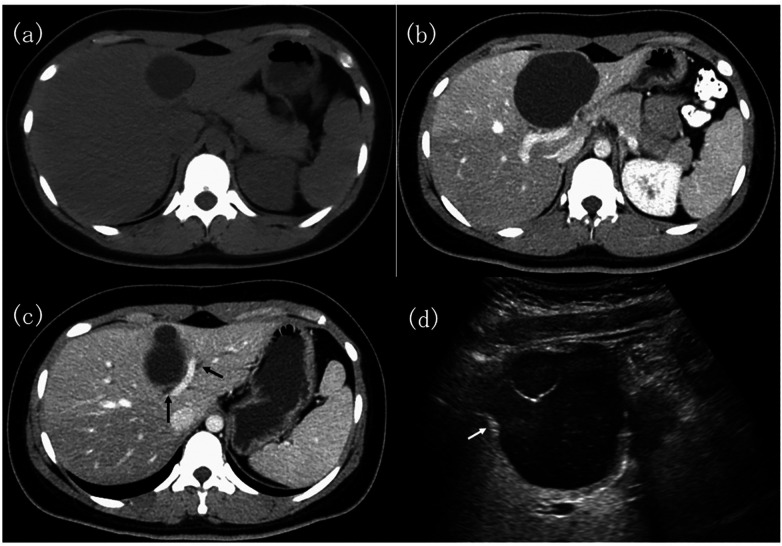
Abdominal computed tomography and ultrasonography findings. **(a)** Non-contrast CT showing a cystic lesion in the medial segment of the liver. **(b)** Contrast-enhanced CT demonstrates a multilocular cystic tumor with calcification of the cyst wall. **(c)** CT image showing dilation of the left hepatic duct and anterior segment bile duct (black arrow). **(d)** Abdominal ultrasonography demonstrates a multilocular cystic lesion with a small hyperechoic mural nodule (white arrow). Reproduced from Moriuchi et al., Cureus (41), licensed under the Creative Commons Attribution–NonCommercial 4.0 (CC BY-NC 4.0) license, Cureus.

On contrast-enhanced CT, MCN-L typically appear as large, well-circumscribed, multiloculated cystic masses with a fibrotic capsule and internal septations, which may enhance after contrast administration. Mural calcifications have been reported in 47%–63% of cases (5, 44, 48). Mural nodules, when present, raise concern for high-grade dysplasia or invasive carcinoma (3, 48). Papillary mural nodules have been consistently associated with carcinomatous change and should be regarded as an important warning feature (18). CT is valuable for surgical planning and assessment of proximity to major vascular and biliary structures but cannot reliably distinguish MCN-L from other cystic liver tumors or exclude malignancy with certainty (1, 48). Representative CT and ultrasonography findings are shown in Figure 2.

### 8.6 Magnetic resonance imaging

**Figure 3:**
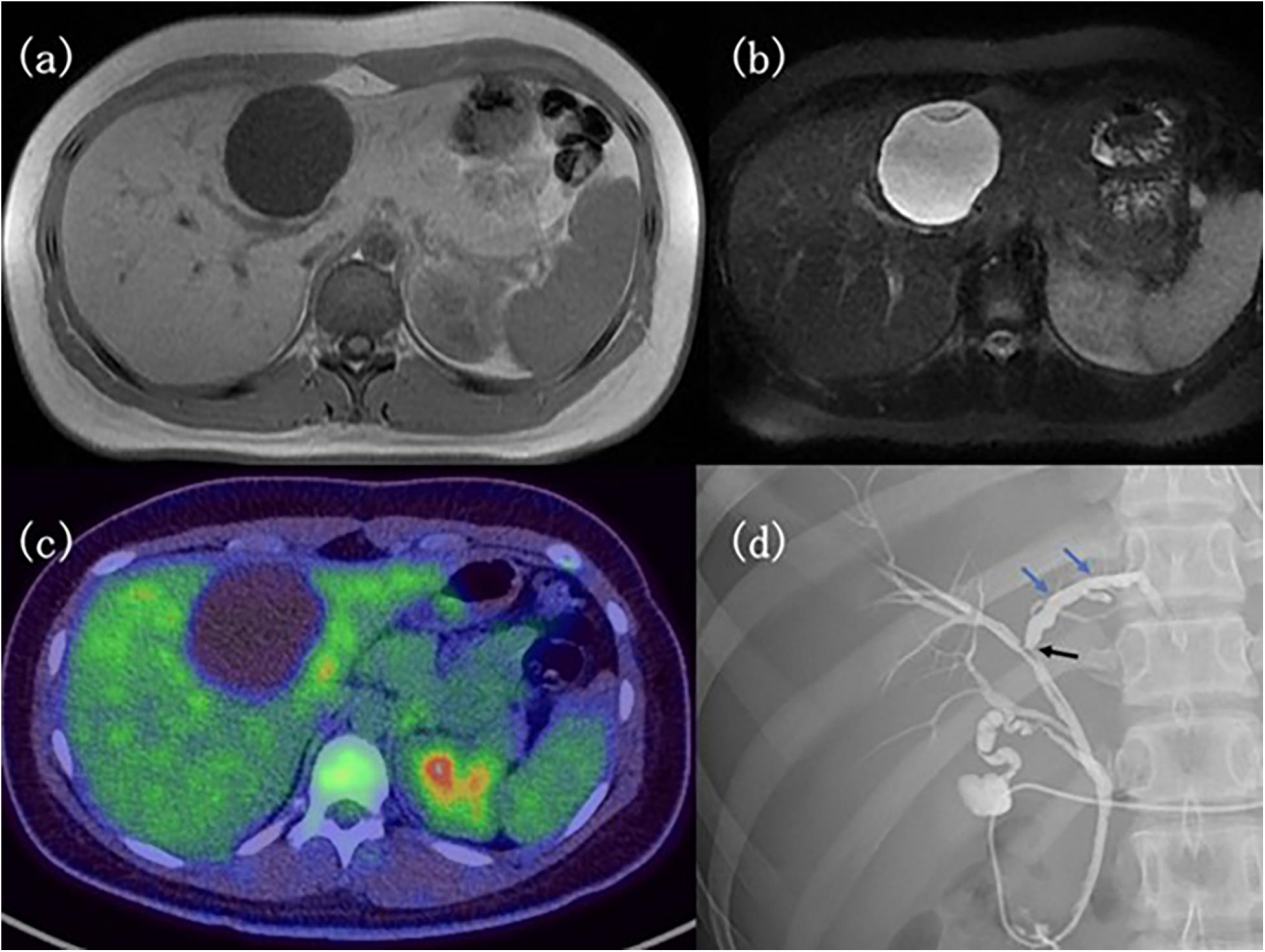
Advanced imaging findings of a hepatic cystic lesion. **(a)** T1-weighted magnetic resonance image showing a hypointense cystic lesion with internal septations. **(b)** T2-weighted magnetic resonance image showing a hyperintense cystic lesion. **(c)** FDG-PET/CT demonstrating no fluorodeoxyglucose uptake within the lesion. **(d)** Endoscopic retrograde cholangiopancreatography showing mild compression and dilatation of the left hepatic and anterior segment bile ducts (arrows). Reproduced from Moriuchi et al., Cureus (41), licensed under the Creative Commons Attribution–NonCommercial 4.0 (CC BY-NC 4.0) license, Cureus.

MRI is the most informative imaging modality for MCN-L. On T1-weighted images, cyst contents typically demonstrate low signal intensity, while T2-weighted images show hyperintense signal. MRI allows superior visualization of internal septations, capsular features, and mural nodules compared with CT (5, 46). MRI with MR cholangiopancreatography (MRCP) is particularly useful in confirming the absence of bile duct communication, supporting a diagnosis of MCN-L, whereas ductal communication favors IPNB (29, 46). Septal enhancement is a highly sensitive MRI feature of MCN-L, although specificity remains limited because similar enhancement may occur in a proportion of simple hepatic cysts (44). Imaging features do not reliably predict histologic grade, and low-grade and high-grade lesions may appear similar on MRI (1, 5). Advanced imaging findings are illustrated in Figure 3. Imaging modality-specific characteristics are summarized in Tables 9, 10.

**Table 9:** Ultrasound and CT characteristics of mucinous cystic neoplasms of the liver based on published series and systematic reviews (5, 6, 12, 14, 44, 48, 50, 51, 57–65).

Imaging modality	Typical morphology	Wall/septal characteristics	Internal components	Features suggesting malignancy	Diagnostic value & limitations
Ultrasound (US)	Large solitary cystic lesion, often multilocular	Thick, uneven wall; echogenic septa	Anechoic or low-level echoes	Mural nodules	Initial modality; operator dependent
Computed tomography (CT)	Multiloculated cystic mass, left-lobe predominance	Fibrotic capsule	Debris or calcifications	Enhancing mural nodules	Useful for surgical planning

MCN-L, mucinous cystic neoplasm of the liver; US, ultrasound; CT, computed tomography.

**Table 10:** MRI and MRCP characteristics of mucinous cystic neoplasms of the liver based on published series and systematic reviews (5, 6, 12, 14, 44, 48, 50, 51, 57–65).

Imaging modality	Typical morphology	Wall/septal characteristics	Internal components	Features suggesting malignancy	Diagnostic value & limitations
MRI	Multilocular cystic lesion	Thick septa; capsular enhancement	Variable T1/T2 signal	Mural nodules; restricted diffusion	Most informative modality
MRCP	Cyst without biliary communication	Septations visible	Limited internal characterization	Biliary communication suggests IPNB	Best for assessing ductal anatomy

MCN-L, mucinous cystic neoplasm of the liver; MRI, magnetic resonance imaging; MRCP, magnetic resonance cholangiopancreatography.

### 8.7 Diagnostic challenges and pitfalls

The primary diagnostic challenge is differentiation of MCN-L from other cystic liver lesions. Misdiagnosis as a benign cyst may lead to incomplete treatment such as fenestration, which is associated with recurrence (3, 14). Radiologic features that increase suspicion include multilocularity, internal septations, wall thickening, and mural nodules; however, no single feature is diagnostic, and invasive carcinoma cannot be excluded with confidence preoperatively (14, 48). A predictive nomogram incorporating contrast enhancement, left-lobe localization, and biliary duct dilatation demonstrated excellent discriminative performance for MCN-L, with a reported C-index of 0.940, substantially improving diagnostic accuracy compared with routine CT or MRI interpretation (56).

**Figure 4:**
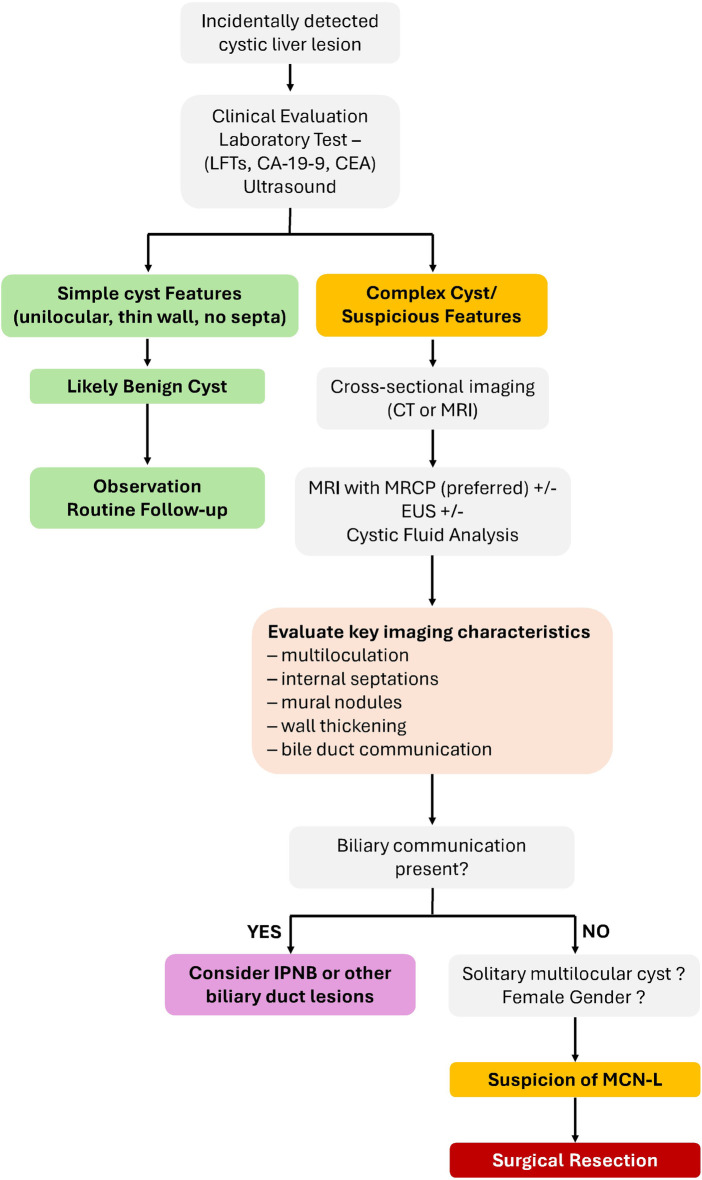
Diagnostic algorithm for the evaluation of cystic liver lesions with consideration of mucinous cystic neoplasms of the liver (MCN-L). Initial assessment typically begins with ultrasound. Lesions demonstrating complex features undergo further characterization with cross-sectional imaging, preferably MRI with MR cholangiopancreatography (MRCP), to evaluate septations, mural nodules, and biliary communication. Suspicion of MCN-L should prompt surgical evaluation.

Imaging modalities serve primarily as a tool for identifying suspicious cystic liver lesions rather than establishing a definitive diagnosis. In summary, MRI with MRCP provides the most comprehensive imaging evaluation for suspected MCN-L, particularly for assessing septal architecture and confirming the absence of biliary communication. Nevertheless, imaging findings alone cannot reliably exclude malignancy or determine histologic grade. Therefore, the presence of suspicious features such as mural nodules, thick septations, or solid components should prompt surgical referral rather than prolonged radiologic surveillance. A proposed diagnostic workflow for the evaluation of cystic liver lesions with consideration of MCN-L is illustrated in Figure 4.

## 9 Role of tumor markers and cyst fluid analysis

Serum tumor markers and cyst fluid analysis have been investigated as adjuncts in the evaluation of mucinous cystic neoplasms of the liver (MCN-L), but their diagnostic value remains limited. Neither approach reliably distinguishes MCN-L from other cystic liver lesions or predicts malignant transformation with sufficient accuracy to guide management decisions (3, 14).

Serum carbohydrate antigen 19-9 (CA 19-9) and carcinoembryonic antigen (CEA) levels may be elevated in some patients with MCN-L; however, these findings are inconsistent and lack specificity. Normal serum levels do not exclude MCN-L or associated malignancy, while elevated values may occur in benign cystic lesions, inflammatory conditions, or biliary obstruction (12, 66). Several studies have also demonstrated no meaningful correlation between serum CA 19-9 or CEA levels and histologic grade, with similar profiles observed in both benign MCN-L and lesions harboring invasive carcinoma (13, 14).

Cyst fluid analysis has also been explored as a diagnostic tool. Measurements of cyst fluid CEA and CA 19-9 concentrations are frequently elevated in MCN-L, but similar elevations have been reported in other benign cystic conditions, resulting in poor specificity (67, 68). In addition, cyst aspiration carries procedural risks, including infection, hemorrhage, bile leak, and potential tumor seeding. Because cyst fluid analysis cannot reliably predict dysplasia grade or invasive carcinoma, routine preoperative aspiration is not recommended when MCN-L is suspected (3, 14).

From a clinical standpoint, tumor markers and cyst fluid analysis have limited utility in the evaluation of suspected MCN-L. Although elevations in serum or cyst fluid CEA and CA 19-9 may occur, these findings lack sufficient specificity to distinguish MCN-L from other cystic liver lesions or to predict malignant transformation. Consequently, normal tumor marker levels should not reassure clinicians or delay definitive treatment when imaging findings raise suspicion for MCN-L. In clinical practice, management decisions should rely primarily on imaging characteristics and overall clinical assessment, with surgical excision remaining the preferred approach when MCN-L cannot be confidently excluded.

## 10. Surgical management

Complete surgical excision is the standard of care for mucinous cystic neoplasms of the liver (MCN-L). Because these lesions possess premalignant potential and invasive carcinoma cannot be reliably excluded preoperatively, definitive resection is recommended once MCN-L is suspected (3, 13, 14). Incomplete procedures such as aspiration, drainage, or fenestration are associated with high recurrence rates and should be avoided (13, 42). The primary goal of surgery is complete tumor removal with negative margins while preserving functional liver parenchyma whenever feasible. Long-term outcomes after complete resection are excellent, and recurrence is rare in the absence of invasive carcinoma (14, 28).

### 10.1 Extent of resection

The extent of liver resection is determined by tumor size, location, relationship to major vascular and biliary structures, and suspicion of invasive disease. Parenchyma-sparing approaches, including non-anatomic wedge resection or segmentectomy, may be appropriate for peripherally located lesions without features suggestive of malignancy (25, 48). In contrast, formal anatomic hepatectomy may be required for centrally located tumors, lesions adjacent to major vascular structures, or when invasive carcinoma is suspected. Intraoperative assessment of mural nodules, cyst wall characteristics, and capsular integrity can assist in guiding surgical decision-making (3, 14).

### 10.2 Minimally invasive approaches

Advances in laparoscopic and robotic liver surgery have expanded the role of minimally invasive approaches for selected patients with MCN-L. Contemporary series have demonstrated that laparoscopic resection can be performed safely in experienced centers with favorable perioperative outcomes and oncologic results comparable to open surgery when appropriate patient selection is applied (25, 69). However, large tumors, lesions suspected of invasive disease, or tumors involving major vascular structures may still require open resection to ensure adequate exposure and margin control (48, 69).

### 10.3 Intraoperative considerations

Careful intraoperative handling is essential to avoid cyst rupture and spillage, which may increase the risk of recurrence. Complete removal of the cyst wall with negative surgical margins is critical to prevent local recurrence (14, 28). Frozen section analysis may assist in confirming the diagnosis and evaluating surgical margins when uncertainty exists, although final histopathologic assessment remains definitive (3, 14).

Lymph node involvement in MCN-L is uncommon and is generally observed only in cases with invasive carcinoma. Routine lymphadenectomy is therefore not recommended unless malignancy is suspected intraoperatively or identified on final pathology (14, 28). When invasive carcinoma is confirmed, management should follow oncologic principles similar to those used for intrahepatic cholangiocarcinoma, including margin assessment and multidisciplinary evaluation (28). In selected cases, intraoperative identification of a clear dissection plane—the peeling sign—may allow parenchyma-sparing excision while maintaining oncologic adequacy.

For surgeons managing cystic liver lesions suspicious for MCN-L, the central principle is complete oncologic excision rather than conservative cyst management. Because malignant transformation cannot be reliably excluded before surgery, aspiration, fenestration, or partial cyst removal should be avoided. The surgical strategy should prioritize complete removal with negative margins while preserving liver parenchyma whenever feasible. Parenchyma-sparing resections may be appropriate for peripheral tumors without features suggestive of invasive disease, whereas centrally located lesions or those with suspicious imaging findings may require formal hepatectomy. When a clear dissection plane is present intraoperatively, techniques such as enucleation guided by the peeling sign may allow safe tumor removal while maintaining adequate oncologic margins. An overview of commonly used surgical strategies for MCN-L, stratified by tumor location and intraoperative findings, is summarized in Table 11.

**Table 11:** Overview of commonly used surgical strategies for mucinous cystic neoplasms of the liver.

Surgical approach	Best clinical setting	Key advantages	Main limitations/risks
Enucleation/parenchyma-sparing excision	Clear dissection plane; positive “peeling sign;” lesion removable intact	Preserves liver parenchyma; minimal tissue loss	Unsafe if dissection plane is unclear; risk of cyst rupture
Non-anatomic wedge resection	Peripheral lesion with achievable margin	Technically simple; adequate margins with limited volume loss	Limited applicability for central or deeply seated lesions
Formal anatomic resection (segmentectomy or lobectomy)	Central lesion; negative peeling sign; suspected invasion	Reliable oncologic clearance	Higher operative burden; greater loss of functional parenchyma
Minimally invasive approach (laparoscopic or robotic)	Selected lesions with favorable location; experienced center	Reduced postoperative pain; faster recovery	Not suitable for all lesions; low threshold for conversion if unsafe

Selection of approach should be guided by tumor location, intraoperative findings, and the ability to achieve complete excision with negative margins. MCN-L, mucinous cystic neoplasm of the liver.

## 11. Intraoperative assessment: the peeling sign and its implications

The “peeling sign” is an intraoperative observation that has emerged as a useful surgical concept in the management of mucinous cystic neoplasms of the liver (MCN-L). It refers to the ability to separate the cystic tumor from the surrounding hepatic parenchyma along a well-defined plane, allowing enucleation or parenchyma-sparing excision without violating the cyst wall (70). This phenomenon reflects the presence of a fibrous capsule and a clear dissection plane between the lesion and adjacent liver tissue. Recognition of this plane may permit limited resection in selected patients while preserving functional liver parenchyma while maintaining oncologic adequacy (70, 71).

When present, the peeling sign enables safe enucleation or limited resection, particularly for large but benign-appearing lesions located away from major vascular or biliary structures. Successful application requires meticulous technique to avoid cyst rupture and ensure complete removal of the cyst wall (70). However, the peeling sign is a technical observation rather than a substitute for oncologic judgment. Surgeons must remain alert for features suggestive of invasive disease, including mural nodules, irregular wall thickening, or disruption of capsular integrity (71).

Conversely, the absence of a clear dissection plane—often termed a negative peeling sign—indicates dense adherence of the lesion to the surrounding liver parenchyma. This may result from chronic inflammation, fibrosis, or invasive tumor growth. In such cases, enucleation is usually unsafe, and formal hepatic resection is required to achieve complete excision with negative margins (70, 71). Attempted limited excision under these circumstances risks cyst rupture, incomplete removal, and recurrence.

Although a negative peeling sign may be encountered more frequently in lesions with high-grade dysplasia or invasive carcinoma, it is not a reliable predictor of histologic grade. Benign lesions may also demonstrate dense adhesions, whereas some dysplastic tumors retain a well-defined dissection plane. Consequently, histologic grade cannot be determined intraoperatively, and definitive diagnosis relies on complete excision followed by pathologic evaluation (14, 28). Clinical series report excellent outcomes when lesions lacking a peeling plane are managed with formal hepatic resection, whereas incomplete excision or attempted enucleation in the absence of a safe dissection plane is associated with increased recurrence risk (13, 42).

**Figure 5:**
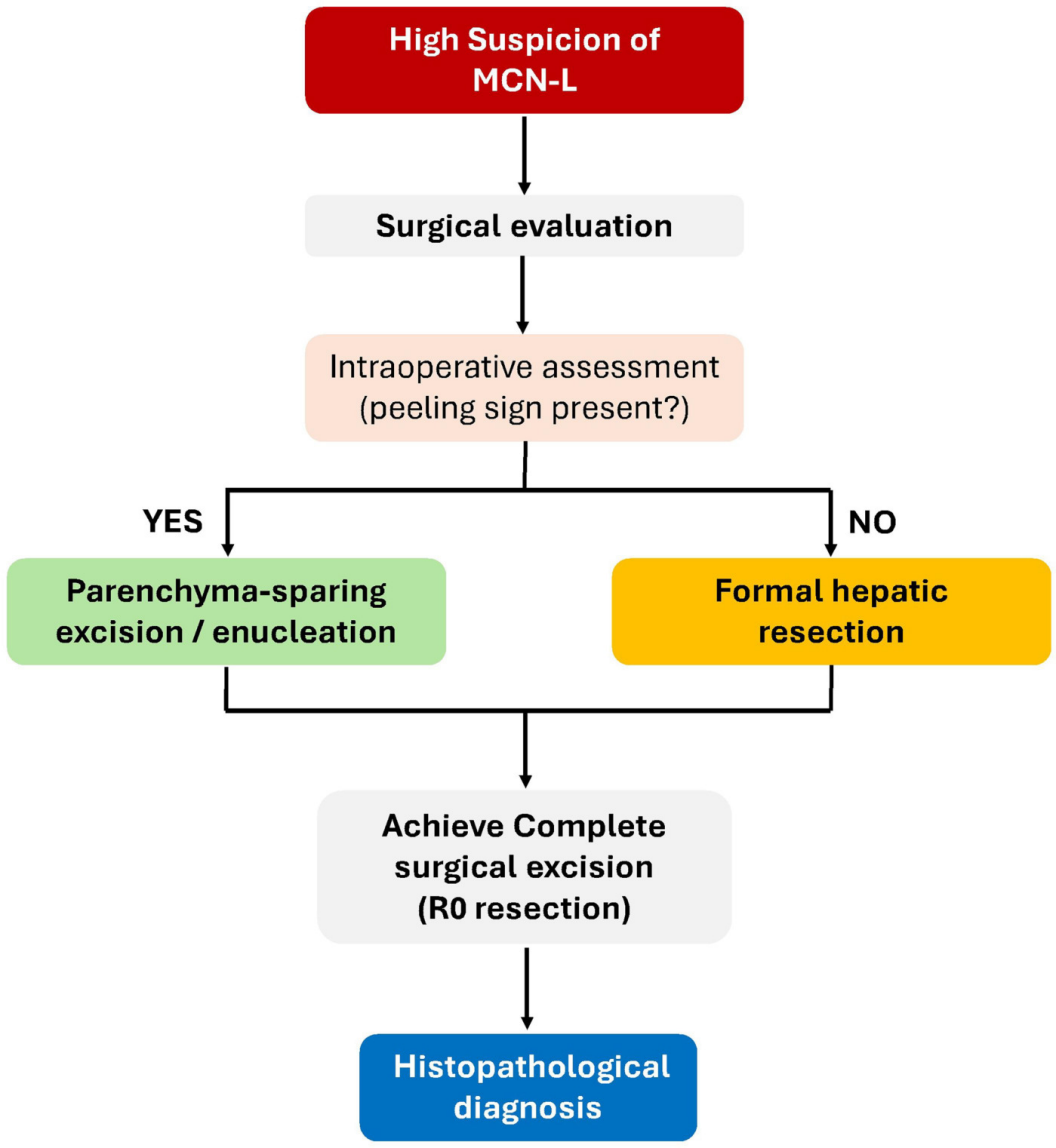
Surgical management pathway for suspected mucinous cystic neoplasms of the liver (MCN-L). Following surgical exploration, intraoperative assessment may reveal the presence of a well-defined dissection plane between the lesion and surrounding hepatic parenchyma, referred to as the peeling sign. When the peeling sign is present, parenchyma-sparing excision or enucleation may be feasible while preserving adjacent liver tissue. In the absence of a clear dissection plane, formal hepatic resection is typically required to achieve complete excision with negative margins. Definitive diagnosis is established by histopathologic confirmation of ovarian-type stroma.

From a practical surgical perspective, the peeling sign should be interpreted primarily as a technical guide to the extent of resection rather than a diagnostic indicator of malignancy. When a clear dissection plane is present and the lesion lacks suspicious features, parenchyma-sparing excision can be performed safely. In contrast, the absence of a peeling plane should prompt conversion to formal hepatic resection to ensure complete oncologic clearance. Surgeons should therefore prioritize margin-negative resection and avoidance of cyst rupture over attempts at maximal parenchymal preservation. A simplified surgical management pathway is illustrated in Figure 5.

## 13. Prognosis and long-term outcomes

The prognosis of mucinous cystic neoplasms of the liver (MCN-L) after complete surgical excision is excellent. Multiple surgical series have reported near-zero recurrence rates following R0 resection of non-invasive MCN-L, with long-term survival approaching that of the general population (13, 14, 28). These favorable outcomes reflect the typically indolent biology of the disease when complete excision is achieved.

Patients with MCN-L containing low-grade or high-grade dysplasia who undergo complete resection generally experience durable cure. Recurrence is rare when negative margins are obtained, and malignant transformation following complete resection has not been convincingly documented in the literature (14, 28, 42). In contrast, incomplete procedures such as cyst drainage or fenestration are associated with a high risk of recurrence, sometimes occurring many years after the initial intervention (13, 42). Recurrent lesions frequently require repeat surgery and may be technically more challenging due to fibrosis and altered anatomy, reinforcing the importance of definitive resection at the time of diagnosis.

Prognosis is less favorable when invasive carcinoma is present. In these cases, outcomes depend on tumor stage, margin status, and lymph node involvement, and clinical behavior resembles that of intrahepatic cholangiocarcinoma arising in non-cirrhotic liver. Nevertheless, patients with invasive carcinoma arising within MCN-L often experience better survival than those with *de novo* cholangiocarcinoma, likely reflecting earlier detection and distinct tumor biology (28, 72).

There is currently no consensus regarding optimal postoperative surveillance following complete resection of non-invasive MCN-L. Given the extremely low recurrence rates, prolonged intensive imaging surveillance is unlikely to be necessary. In contrast, patients with invasive carcinoma should undergo structured follow-up consistent with surveillance protocols used for intrahepatic cholangiocarcinoma (28).

The most important determinant of outcome is complete surgical excision at the time MCN-L is identified. Patients with non-invasive disease who undergo R0 resection can generally be considered cured and do not require intensive long-term surveillance. Conversely, incomplete procedures predispose to recurrence and often necessitate more complex reoperations. When invasive carcinoma is identified, prognosis and postoperative management should follow oncologic principles used for intrahepatic cholangiocarcinoma, including structured surveillance and multidisciplinary evaluation.

## 14. Controversies and future directions

Despite advances in the understanding of mucinous cystic neoplasms of the liver (MCN-L), key challenges remain in preoperative diagnosis, surgical strategy, minimally invasive approaches, and postoperative surveillance.

Preoperative differentiation from other cystic liver lesions remains unreliable, as imaging, tumor markers, and cyst fluid analysis cannot consistently determine histologic grade or exclude invasive carcinoma. Consequently, management decisions continue to rely on clinical suspicion rather than definitive diagnosis (1, 3, 44, 55).

Surgical strategy remains debated. While parenchyma-sparing approaches may be feasible in selected cases with a clear dissection plane (peeling sign), concerns regarding incomplete excision and recurrence persist. Formal hepatic resection remains the preferred approach in lesions with uncertain features or oncologic concern (14, 70).

Minimally invasive liver surgery has demonstrated favorable perioperative outcomes but lacks robust long-term oncologic validation, particularly for large or complex lesions (25, 69). Postoperative surveillance is also not standardized, with unclear recommendations for patients with high-grade dysplasia or uncertain margins (28).

Future efforts should focus on improving risk stratification and understanding tumor biology. Radiomics and machine-learning models show promise in imaging-based diagnosis, while molecular profiling may identify biomarkers of progression. Multicenter prospective registries will be essential to address current evidence gaps. The key controversies and proposed future research priorities in MCN-L are summarized in Table 12.

**Table 12:** Key controversies and future research priorities in MCN-L.

Domain	Current limitation/controversy	Clinical implication	Future direction
Preoperative diagnosis	Imaging, tumor markers, and cyst fluid lack sensitivity/specificity for grade and invasion. (1, 3, 44, 55)	Surgical decisions based on suspicion rather than confirmed diagnosis	Radiomics and machine-learning models to improve diagnostic accuracy
Extent of resection	Debate between parenchyma-sparing approaches vs. formal hepatectomy. (14, 70)	Risk of incomplete excision and recurrence with limited surgery	Prospective studies to define selection criteria for safe parenchyma-sparing surgery
Minimally invasive surgery	Limited long-term oncologic outcome data. (25, 69)	Uncertainty in applying MIS to large or complex lesions	Multicenter outcome studies comparing MIS vs. open approaches
Postoperative surveillance	No standardized follow-up protocols.	Potential over- or under-surveillance	Development of risk-adapted surveillance guidelines
Tumor biology	Poor understanding of molecular drivers of progression	Inability to predict malignant transformation preoperatively	Molecular profiling to identify biomarkers (e.g., KRAS-related pathways)
Evidence base	Predominantly retrospective, surgical-only cohorts	Selection bias limits generalizability	Multicenter prospective registries and collaborative datasets

## 15. Conclusions

Mucinous cystic neoplasms of the liver (MCN-L) are distinct premalignant cystic tumors defined by ovarian-type stroma and characterized by an inherent risk of malignant transformation. The key clinical challenge remains the inability to reliably differentiate these lesions or determine histologic grade preoperatively. Management therefore prioritizes oncologic certainty over diagnostic ambiguity. Complete surgical excision provides both definitive diagnosis and cure, with parenchyma-sparing approaches reserved for carefully selected cases where oncologic principles are not compromised. MCN-L should be treated as a surgical disease once suspected, rather than subjected to prolonged diagnostic evaluation. Future progress will depend on advances in imaging-based risk stratification, molecular profiling, and collaborative prospective data. Until reliable noninvasive diagnostic tools are available, early recognition and definitive surgical excision remain the most effective strategy to prevent recurrence and malignant transformation.
